# AlignHUSH: Alignment of HMMs using structure and hydrophobicity information

**DOI:** 10.1186/1471-2105-12-275

**Published:** 2011-07-05

**Authors:** Oruganty Krishnadev, Narayanaswamy Srinivasan

**Affiliations:** 1 Molecular Biophysics Unit Indian Institute of Science, Bangalore 560012, India; 2 A318, Biochemistry and Molecular Biology Department, 120 East Green Street, Athens, GA 30602, USA

## Abstract

**Background:**

Sensitive remote homology detection and accurate alignments especially in the midnight zone of sequence similarity are needed for better function annotation and structural modeling of proteins. An algorithm, AlignHUSH for HMM-HMM alignment has been developed which is capable of recognizing distantly related domain families The method uses structural information, in the form of predicted secondary structure probabilities, and hydrophobicity of amino acids to align HMMs of two sets of aligned sequences. The effect of using adjoining column(s) information has also been investigated and is found to increase the sensitivity of HMM-HMM alignments and remote homology detection.

**Results:**

We have assessed the performance of AlignHUSH using known evolutionary relationships available in SCOP. AlignHUSH performs better than the best HMM-HMM alignment methods and is observed to be even more sensitive at higher error rates. Accuracy of the alignments obtained using AlignHUSH has been assessed using the structure-based alignments available in BaliBASE. The alignment length and the alignment quality are found to be appropriate for homology modeling and function annotation. The alignment accuracy is found to be comparable to existing methods for profile-profile alignments.

**Conclusions:**

A new method to align HMMs has been developed and is shown to have better sensitivity at error rates of 10% and above when compared to other available programs. The proposed method could effectively aid obtaining clues to functions of proteins of yet unknown function.

A web-server incorporating the AlignHUSH method is available at http://crick.mbu.iisc.ernet.in/~alignhush/

## Background

Alignment between sequences is useful and ubiquitous in bioinformatics [1]. Many of the advances made in the field of bioinformatics can be attributed to advances in alignment of sequences. The performance of homology-based structural modeling methods in CASP over last several years is strongly correlated to the accuracy of the alignment between template and the target [2]. Alignments are also routinely generated for effective identification of remote homologues leading to function annotation of newly discovered proteins from genome sequence data [3,4]. The explosion of sequence data from genome sequencing projects has exposed the limitation of current methods to recognize homologues in the twilight region (<30% sequence identity) and beyond (the midnight region of sequence similarity).

It was found quite early that profile methods, such as PSI-BLAST [5,6] can be more sensitive and accurate than single sequence-based methods. The starting point for deriving various kinds of profiles such as Position Specific Scoring Matrices (PSSMs) and Hidden Markov Models (HMMs) is an alignment of sequences of homologous proteins or protein domains. The commonly used profiles are the PSI-BLAST generated PSSMs derived from query dependent alignments [5,7], Multiple Sequence Alignment (MSA) based position specific gap penalty profiles [8] and HMMs [9,10]. In general, HMMs have been shown to be more sensitive than other profile based sequence-profile search methods [11]. This is usually attributed to the ability of HMMs to parameterize position specific gaps. The use of dirichlet mixture priors in estimation of amino acid probabilities in a MSA column could also be a reason for the success of HMMs [12].

The profile in general is a representation of conservation of amino acids at a given position in the MSA [13,14]. Thus, it was believed that aligning two profiles will lead to better sensitivity and alignment accuracy as only the patterns in extent of conservation will be taken into consideration.

Various methods have been developed for the alignment of profiles, which differ in the input profile type and also the scoring scheme used for the alignment of columns in the two profiles. For instance, in the MSA derived profile/PSSM based profile-profile alignment methods, COMPASS [8], SALIGN [15], COACH [16], and others [17] have been shown to give better sensitivity (~ 30 - 35%) [18] than simple sequence profile search methods such as PSI-BLAST and HMMER2.0 (sensitivity around 20% at superfamily level) [18]. Some of the recent developments using the PSI-BLAST programs such as MulPSSM [19] and Cascade BLAST [20] have increased sensitivity of PSI-BLAST methods by a few percentage points, but they may not be suited for homology based structural modeling since they depend on PSI-BLAST for alignments, which has modest alignment accuracy in the midnight region [21,22]. Another recent development [23] has increased the sequence-profile based sensitivity is HMMER3.0 which has pushed sensitivity at the SCOP superfamily level to around 30% at 10% error rate.

Two recent methods based on HMM - HMM alignment [18,24] have shown that HMM based profile-profile alignment methods are more sensitive than PSSM-PSSM alignment methods, although at least one recent development in alignment of MSA's [25] seems to give better sensitivity than the HMM based methods at 50% error rate. The HMM-HMM alignment in a general case is thought of as maximizing the probability of co-occurrence of amino acids along an alignment path between the emission states of the two HMMs [18]. This can be achieved by different combinations of pairwise state transitions as described in the Profile Comparer (PRC) method [24]. The most important parameter which determines the sensitivity of a method is the column discrimination score, and both PRC [24] and HHSearch [18] use a log sum of odds score for aligning HMM match states (which correspond to a column in MSA). The alignment accuracy (calculated using a structural measure used for evaluating predicted structures) is generally high (>85%) for both methods, even if the alignment length is usually quite low for HMMs of distantly related domain families (belonging to the same SCOP superfamily but different SCOP families) [22]. The increase in both sensitivity and alignment accuracy is also in part due to the use of secondary structure information in the HHSearch alignment procedure and such an increase in remote homology detection rates by the use of secondary structure information has been reported earlier [26].

Use of secondary structure information alone (either predicted or actual) can lead to increase in false positive rate since there could be many proteins with similar sequence of helices and β-strands, but different 3-D topology. The algorithm presented in this paper takes into account the residue conservation, secondary structure propensity and hydrophobicity of not only the columns being aligned, but also the adjacent columns, thus enabling it to differentiate HMMs with similar secondary structure but different topology.

## Methods

The AlignHUSH procedure has been developed to detect remote homologs more effectively than the currently existing tools without compromising on the alignment accuracy. The objective of the current work was to develop a generally applicable algorithm without the use of explicit structural information to align two sequence-based HMMs. The sequence conservation information based profiles currently used for remote homology detection are inadequate for detection of very remote homologs since it is known that the sequence conservation is not seen in such cases of remote homology. The structure is still seen to be conserved and hence use of structural information can lead to an improvement in the remote homology detection [18]. The use of structural information may lead to many relationships being incorrectly found as significant matches in a search since there may not be a robust method to distinguish between related and unrelated sequence of secondary structures. One way to distinguish such cases would be to find the packing of the secondary structures, but such information can be derived only from an analysis of the 3-D structure. An approximation to the packing can be derived from the hydrophobicity pattern since it is known that the core of any protein is composed of hydrophobic residues. Such an approach has been shown to improve remote homology detection in sequence-profile alignments recently [27]. Thus, in the AlignHUSH procedure a contribution to the score is derived from explicit hydrophobicity calculations which might enable the detection of remote homologues with finer detail.

Another fact that has been exploited in the development of the AlignHUSH procedure is the observation that conservation of residues occurs over a stretch of residues. Thus, adding the contribution from neighboring columns should enable better differentiation than by using information from a single column. In case of a match between homologous patches, the score per column is enhanced but in unrelated patches, it is expected that the effect will be on average cancel out and the score for such patches will remain largely unchanged. Thus, use of neighbor information is expected to increase the separation between the scores for homologous matches and non-homologous matches which should enhance the difference in scores between the unrelated and related profiles.

In the next few sections, the incorporation of these ideas into the algorithm will be described in detail, followed by assessment based on the SCOP database.

### Datasets used

The dataset of HMMs used in the analysis was obtained from the Superfamily database [28] which provides HMMs and PSSMs of SCOP database. SCOP 1.69 [29,30] has been used throughout the analysis for parametrization. Out of the 10,894 HMMs present in the database corresponding to 2829 SCOP families, one HMM has been selected randomly from each family. This resulted in a total of 2829 families which will henceforth be called the 'full SCOP 1.69 dataset'. The latest SCOP database (version 1.75) profiles from Superfamily database were also used to determine the sensitivity and specificity. The HHSearch compatible HMMs for the Superfamily database (version 1.75) were downloaded from HHSearch website ftp://toolkit.lmb.uni-muenchen.de/HHsearch/databases/. From a total 13921 profiles, extraction of single profile per family as performed for SCOP 1.69 database lead to a dataset of 3464 profiles which were used for comparison of the performance of three methods.

The true relationships in this work have been defined as two profiles corresponding to two SCOP families that belong to the same SCOP Superfamily. Any two profiles belonging to SCOP families that are present in different classes have been deemed as unrelated (false positives in a search). The SCOP families belonging to two different folds in the same class are ignored. In the next section, the procedure used to incorporate additional information to HMMs is described.

### Format of HMMs in AlignHUSH procedure

The AlignHUSH procedure uses information which is not present in the HMMs generated by HMMER/SAM packages. Hence, the input HMM was re-encoded to AlignHUSH format and this is explained in detail in the subsequent sections.

#### Gap penalties

HMMER2.0 uses the plan7 architecture of HMMs, which has been well documented (refer to the User Guide of HMMER2.0). The architecture results in three 'states' at each 'node' (a node corresponds roughly to a column of a MSA). The states can be either M (match), I (insert), or D (delete) states. Each of the match/delete states correspond exactly to one column in the MSA from which the HMM is derived. Multiple columns of MSA can lead to a single Insert state. The Match - Match transition probability is close to 1 in most nodes. Where the probability of occurrence of inserts is high, the transition from a previous match state to the current insert state is also high. This leads to a position-specific gap penalty which is one of the reasons why HMMs are more accurate and sensitive than PSSM-based methods which usually have uniform gap penalties. The alignment between profiles is usually thought of as optimal match of two conservation patterns. Hence, the information on the probability of insert at a site is important but, the composition of insert states is not important. Thus, the insert emission line is discarded in the HMMs for use in AlignHUSH.

#### Scores for emission of amino acids at each match state

The conservation of amino acids at a position in a MSA is not sufficient to discriminate between related and unrelated patterns. Thus, to enhance the information content of a column in the MSA, information on the hydrophobicity and predicted secondary structure scores are added to a column of the MSA. The HMMER suite of programs stores the match emission probabilities as log odds scores. These scores are first converted into probabilities using the equations specified in HMMER UserGuide. The probabilities are modified and stored as ratios of probability of amino acid to square root of background probability of that amino acid (this step makes it easy to calculate sum of log odds ratios later). The explanation for this unusual step will be made clear when alignment procedure is explained.

Since AlignHUSH uses hydrophobicity and secondary structure at each position, these values/features are incorporated as emission probabilities set. The hydrophobicity of each state is calculated as the product of probabilities of amino acids and the Kyte-Doolittle hydropathy values [31] for the respective amino acids. For the generation of secondary structure scores, secondary structures of each of the sequences in the MSA have been predicted using PSIPRED [32] and the frequency of occurrence of each of the secondary structures in a column of the MSA has been calculated. Since each column in the MSA corresponds to either match state or an insert state, the probabilities of secondary structures occurring in a MSA column can be transferred to either a match state or an insert state. The frequencies for each secondary structural state, for every match position is stored and used for calculating the secondary structure based score.

### Alignment scheme

The alignment of profiles can either be global or local. Most profile-profile methods use local alignments since it is thought that only highly conserved amino acid motifs are conserved across families in a SCOP superfamily. This view is appropriate when conservation of amino acids is used as a measure of relatedness. But, it is known from structural classification databases that even if sequence patterns are not conserved, structure is conserved in such sequence fragments. Since the objective of the AlignHUSH method is to recognize remote homologs it is imperative that the alignment length cover at least the structurally similar core of the two families in consideration. This can be achieved if local alignments are constructed and parameters are optimized to allow for the local alignment to cover most of the homologous regions. The Viterbi algorithm has been used for local alignments of profile-profile matches, and the equations used closely follows the procedure of HHSearch [18] and are reproduced in this manuscript. The procedure is briefly described in the next few subsections.

The Viterbi procedure breaks down the goal of aligning two profiles into a series of local optimizations. Initially as no alignment is made yet, the score is set to zero. Throughout this section, one of the two profiles is defined as P(1....n), which is a profile P with 'n' match states, and the other profile is represented by Q (1....m) which is the profile Q with 'm' match states. P(i) means the ith column of profile P and P(ia) means the a^th ^amino acid in the i^th ^column in profile P. The notation for state transition probabilities is derived on similar lines. For example, for a state transition from Match state at i-1^th ^position in profile P to insert state at i^th ^position in profile P is given as P(M->M)_i-1_.

The alignment between two profiles is thought of as a pair-HMM following the suggestions made in HHSearch procedure [18]. According to the HHSearch procedure, among the nine pair-HMM states, five states MM, MI, IM, DG, and GD are sufficient for describing the profile-profile alignment, where M means 'match', D means delete, G means gap and I means insert in a profile. The Viterbi procedure starts by setting up five matrices for each of the pair-HMM states. The total score till Pi and Qj are aligned is given by the variable Sij (one for each of the five matrices) which is maximized in the Viterbi procedure. The equations for aligning the profiles closely follows the procedure suggested in HHSearch [18] and are given in detail as equations 1a - 1e. The difference between the HHSearch procedure and AlignHUSH lies in the calculation of the score for alignment of two match positions, which is described below(1a)(1b)(1c)(1d)(1e)

In the AlignHUSH procedure, the match between the columns consists of three scores, the hydrophobic score, the conservation score and the secondary structure score. The conservation score is taken from the work of Soding on HHSearch [18], and is given as a log-sum of odds score (equation 2) where b_a _refers to the background frequency of amino acid 'a'. The conservation patterns are usually observed in short motifs, and not in isolation, and hence in AlignHUSH procedure, the conservation score is taken over a window of a few columns. Thus the conservation score at position (i,j) is the sum of conservation scores over a window. Optimization using sensitivity as a criterion revealed that a window size of 5 gives the best results and this has been used throughout the analysis (indicated in equation 1). The hydrophobic score and the secondary structure score are likewise derived (equations 3 and 4 given below) and the window size for each score is determined separately. In equation 3, Hx and Hy refer to the hydrophobicity of a column at position x in first HMM and position y in second HMM. Similarly, the f(x)_s _term is the frequency of occurrence of secondary structure 's' at position x in first HMM, and likewise for the second HMM (at position 'y'). The Lss term in the equation 4, is taken from a substitution table of secondary structural elements that was derived during the AlignHUSH development. The substitution table was derived by calculating the frequency of substitution of one secondary structure (Helix, Sheet, Loop), with another in structural alignment of SCOP family sequences.(2)(3)(4)(5)

The three scores combined with their respective weightage given to each term gives the final column match score (equation 5) that is used to calculate the column match score (Wc, Wh and Ws are the weights given to the conservation core, hydrophobic score and the secondary structure score). In addition to the weights given to the column match score parameters, an additional weightage term is used for gap penalties (the log terms in equations 1a to 1e. This has been done to ensure that the use of neighbor information and additional information does not make the Mij term much larger than the gap penalty terms which could lead to a long alignment between unrelated profiles. Traceback pointers are stored at each position to allow for the alignment to be re-generated later. The traceback starts from the element in the MM matrix where the score is highest, which is also the score determined for the alignment between the profiles. In the next section the definitions of sensitivity and statistical assessment of the method is described in more detail.

### Statistical Assessment and sensitivity and error rate analysis

Statistical assessment of similarity between proteins is usually expressed as E-values. The E-value as defined by [33,34] is the number of random alignments (or profiles) which can be expected to give a score better than the score that is observed between two proteins. If the number is very low, then the alignment score is very significant. The E-value parameters for local ungapped alignments are well understood, but heuristic measures are used for the case of gapped local alignments. The assessment of statistical significance in case of HMMs is more complicated and several methods have been proposed based on whether the alignment has been generated using Viterbi algorithm or the full forward algorithm [9,35]. In order to decrease the bias due to the length of profile or composition of the profile, a per-family assessment of significance is better suited than an estimation of database specific parameters. For assessment of statistical significance, the distribution followed by alignment scores of random profiles is required. An approximation to alignment to random profiles can be obtained by aligning each query HMM to a large randomly selected profile database. The scores obtained using the random database of profiles is used to fit the E-value parameters and these are used to report E-values in searches.

The definition of homologous and non-homologous families is subjective in several cases, with various classification schemes following different criteria for determining homology. Since the HMMs are derived based on a SCOP based classification, homology as proposed in SCOP has been used. Thus, if two HMMs belong to same SCOP superfamily, then they are considered homologous. HMMs belonging to the different SCOP classes were considered clearly non-homologous Two HMMs belonging to the same SCOP fold but different SCOP superfamilies, or two HMMs belonging to different folds in the same class are ignored and not evaluated as either true positives or false positives. The sensitivity is defined as TP/(TP+FN) and error rate as FP/(FP+TP), where TP stands for true positives, FN stands for false negatives and FP stands for false positives.

The comparison of sensitivity has been made with two other methods namely, PRC (version 1.5.6) and HHSearch (version 1.5.0 with SCOP1.69 and 1.5.1 with SCOP 1.75 profiles). Since PRC accepts HMMs in HMMER format, no conversion of HMM format was needed to search in the SCOP database of HMMs provided by Superfamily database. The HMMs (for SCOP1.69 version) downloaded from the Superfamily database were converted into the HHSearch format by using the 'hhmake' program and the predicted secondary structure was added using the HHSearch tool 'addpsipred.pl' in order to use secondary structure scores in HHSearch. For the latest SCOP (1.75 version) database, the profiles corresponding to latest SCOP were downloaded from HHSearch web site in HHSearch compatible format. The calibration of each of the HMMs in the SCOP1.69 and the latest SCOP database was done by searching in the calibration database provided with the respective HHSearch programs. Both PRC and HHSearch were used with an E-value of 10 as default, and in case of HHsearch, an option to print at least 100 hits was used to estimate correctly the number of profiles found as false positives for a query.

### Assessment of Alignment Accuracy

The assessment of alignment accuracy especially for remotely related proteins is practically not feasible since the evolutionary history of any protein cannot be determined with high accuracy. Currently, there are no methods available which can provide the evolutionary path through which two proteins might have diverged from an ancestor protein. The alignment is a statistical approximation to this path and its accuracy cannot as such be measured reliably. In the absence of such 'gold standard' alignments, careful approximations must be made to derive a set of reference alignments against which the accuracy can be gauged. At present, the structure based alignments are considered the 'gold standard' especially if manual curation has been made to refine small misaligned regions. There are several such databases, out of which the BaliBASE database [36] was chosen since it provides alignments based on sequence identity cutoffs (as low as < 20%), which enables the assessment of alignment accuracy for remotely related proteins.

The BaliBASE database provides manually curated structure-based alignments which have been pruned to ensure that the secondary structure elements align well, and care is also taken to ensure that functional residues are always aligned. Thus, the alignments present in the BaliBASE database are considered as the 'gold standard' of alignments. The comparison of alignments (from SCOP 1.69 dataset) generated using AlignHUSH, PRC and HHSearch with reference alignments in this database was done and the correctly aligned columns in each case were determined. The procedure followed was to take the RV11, RV12, RV20 and RV30 subsets of the BaliBASE 3.0 database, which give alignment between proteins with less than 30% sequence identity. There were 745 pairs of proteins (number of unique proteins being 317), which have less than 30% sequence identity and which are also in different SCOP families in the same SCOP superfamily. The sequences of the 317 proteins were aligned to its cognate SCOP family using the hmmalign program in the HMMER suite of programs. This step might introduce inaccuracies, since the sequence-profile alignment is not 100% accurate. The profile-profile alignment between two SCOP families is taken from each of the three methods, and a sequence - sequence alignment of remotely related proteins is generated by combining the two sequence-profile alignments with the profile-profile alignment. For each test alignment (generated by AlignHUSH, PRC and HHSearch), the region of overlap with the reference alignment was taken and the proportion of correctly aligned residues was calculated (ratio of correctly aligned columns to total aligned columns present in both reference alignment and the test alignment).

## Results

### Comparison of AlignHUSH to other HMM profile alignment methods

**Figure 1:**
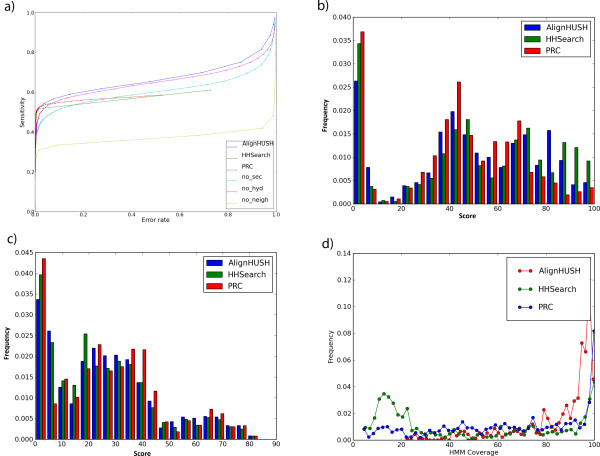
**Comparison of performance of AlignHUSH method to HHSearch and PRC**. **A) **The sensitivity and error rate values for both AlignHUSH and HHSearch are plotted in this figure. The sensitivity of AlignHUSH is better than HHSearch or PRC at almost all error rates. The 'no_sec', 'no_hyd' and 'no_neigh' are variants of AlignHUSH procedure without use of secondary structure, hydrophobic and neighboring column information respectively. **B) **Alignment accuracy of the three methods that have been examined in detail in the main text. The alignment accuracy given in this plot corresponds to the 'developer score' defined in the main text. The three methods are comparable as far as the accuracy using developer score is concerned. **C) **The alignment accuracy of the three methods using the 'modeller score' defined in the main text. The performance of AlignHUSH is slightly better than that of HHSearch and PRC. HHSearch generated alignments tend to be very short and hence HHSearch has a low value for 'modeller score' alignment accuracy. **D) **The length of the query HMM covered by the alignment is plotted for the alignment between homologous families (two SCOP families belonging to the same SCOP superfamily). The coverage of query HMM is greater in case of AlignHUSH than HHSearch which indicates that AlignHUSH generated alignments are more informative for function annotation, since they cover almost the entire homologous region. The alignment length coverage is very similar between the PRC generated alignments and AlignHUSH generated alignments.

The plots in Figure 1a, show that when using the same query and database, AlignHUSH (when using all three parameters with neighbor information) performs better than HHSearch and PRC at all error rates. The sensitivity at 5% and 10% error rate for a number of other profile-profile alignment methods is given in Table 1. The data in the Figure 1a and the Table 1 demonstrate that the sensitivity of AlignHUSH, especially when all three scoring parameters are used, is slightly higher than all the currently existing methods. For comparison, the sensitivity of AlignHUSH at 10% error rate is 57%, whereas the sensitivity of HHSearch is 51% and that of PRC is 54%. The comparison of HHSearch and PRC to other methods has been made in an independent assessment reported by Sadrayev and Grishin [22] (Table 1) that reveals that HHSearch and PRC perform better than other profile-profile alignment methods. The definition of true positives in this work is deliberately restricted to families within a SCOP superfamily since such relationships can be considered evolutionarily related with high confidence. SCOP families belonging to different folds could still have homology and hence an assessment of sensitivity and error-rate was performed with families within same fold considered as true positives and the results are similar to that observed in the case of SCOP superfamily analysis (sensitivity and error-rate plots are provided in Additional File 1).

**Table 1:** Sensitivity at three levels of error-rate for a few profile-profile search methods.

Method	Sensitivity at 5% error rate	Sensitivity at 10% error rate	Sensitivity at 50% error rate	Source
AlignHUSH	52.5%	57.2% (58.5%*)	66.8%	Current work

HHSearch (with SS)	51%	51.3% (48.8%^§^, 54.2%*)	56%	Current work and Soding, 2005 [18]

HHSearch (no SS)	NA	46.7%	NA	Soding, 2005 [18]

PRC	53.2%	54.4% (53.2%*)	58.6%	Current work

PROCAIN	NA	52% ^#^	~60% ^#^	Wang et al, 2009 [25]

PROF_SIM	NA	24.9%	NA	Soding, 2005 [18]

COMPASS	NA	34.0%	NA	Soding, 2005 [18]

The sensitivity values at three levels of error rates for some of the most commonly used profile-profile search methods. The source of each value is given in the last column. Note that the datasets used and the definitions used for true positives can differ from one source to another and hence the values are not comparable across different sources.

"#": The values given for PROCAIN are extracted from Fig 2b of Wang et al [25] where the definition of true positives and false positives is similar to that used in the current work. "§": Value reported from Soding, 2005 [18]. "*": The values reported with asterisk are for the comparison using the latest SCOP release. For HHSearch the value reported is for the latest version of the program.

The use of neighbor information has lead to a significant increase in the sensitivity at all error rates as demonstrated by the plots in Figure 1a, which shows an increase of around 20% when such information is used. The use of secondary structure information has also lead to an increase as can be seen from comparison of AlignHUSH with no secondary structure scoring (51.6% sensitivity at 10% error rate) and AlignHUSH (57.2% sensitivity at 10% error rate) in Figure 1a. The use of hydrophobicity information also leads to a modest increase in sensitivity of AlignHUSH procedure. The major difference between AlignHUSH and the other methods is the use of neighbour information and this parameter has lead to the increase seen in the sensitivity of AlignHUSH. The assessment of sensitivity of the three methods AlignHUSH, HHSearch and PRC was also done for the latest SCOP database version 1.75 profiles (Additional File 2). The sensitivity of HHSearch in the latest SCOP database shows an increase of 2%, AlignHUSH shows an increase of 1% and PRC shows a decrease of 1% suggesting that the sensitivity values for each method could vary with changes in the underlying database.

The alignment accuracy of the three methods compared in this paper was calculated as two different values that give slightly different views on the alignment accuracy. The first value is the 'developer score' which is the ratio between the number of correctly aligned columns (with respect to a reference alignment) and the alignment region. The second score is the 'modeler score' that gives the ratio of correctly aligned columns and the length of the reference alignment. The two values can be thought of loosely as the specificity of the alignment and the sensitivity of correct alignment. The two alignment accuracy values for each of the three methods tested (viz AlignHUSH, PRC and HHSearch) are given in Figure 1b and 1c. The average alignment accuracy for the three methods when the developer score is calculated is AlignHUSH (49.4), PRC (42.9) and HHSearch (51.2). The developer score for HHsearch is higher than that of PRC or AlignHUSH, but this could be due to the short alignments generated by HHSearch (comparison of length of alignments is given in Figure 1d). The 'modeller score' on the other hand is a measure of how much of the alignable region has been correctly aligned by a method. Thus, the modeler score is of great interest from a user's perspective. The values for the three methods are AlignHUSH (26.6), PRC (27.4) and HHSearch (24.6). Thus, the AlignHUSH procedure compares favorably to the best performing methods as far as alignment accuracy is concerned. The plots in Figure 1d suggest that the length of alignment is usually as long as the length of the query profile in case of AlignHUSH alignments which would be useful for functional and structural studies.

**Figure 2:**
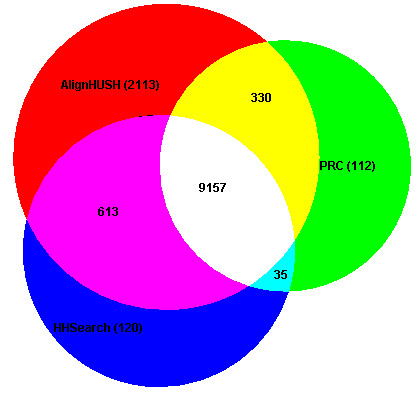
**Figure showing the overlap between the related SCOP families found as hits with E-values better than 10 for the three methods studied in this paper**. The numbers given along with the name of the method are the true relationships found uniquely using the method. The numbers given in the overlap regions are the relationships found using one or more of the methods. Figure generated using the web-tool from http://www.cs.kent.ac.uk/people/staff/pjr/EulerVennCircles/EulerVennApplet.html.

The values obtained for sensitivity at 10% error rate for the three methods studied, are useful as a guide for automated annotation efforts. For detailed analysis of a few families, it is usually preferable to detect as many remote homologs as possible, which can then be pruned based on additional biological data (for example information on active site residues). For such efforts, the AlignHUSH procedure can be more useful than HHSearch or PRC since it provides better sensitivity at higher error rates (Figure 1a). Moreover, from a user perspective, it is always advisable to use as many methods as available to increase the chances of recognizing meaningful remote homologues, since none of the methods recognize all the available remote homologues. An analysis of the overlap between various methods can thus be indicative of the relative merits and demerits of the use of multiple methods. Such an analysis has been performed in this paper, where the pairwise remote homologs identified between SCOP families (within the same SCOP superfamily), are considered (given in Figure 2) at a low E-value threshold of 10 for each of the methods. The most significant point that emerges from the figure is the high degree of overlap between HHSearch and PRC results. On the other hand, the number of hits found unique to AlignHUSH procedure is very large and suggests that the scoring scheme of AlignHUSH is sufficiently different from that of the other two methods. Thus, even though the sensitivity values considered for the whole dataset is marginally better, the biological value added due to the use of AlignHUSH can be enormous as far as remote homology detection is concerned considering the fact that AlignHUSH takes roughly only twice as long as HHSearch to search the same number of profiles (data not shown). This point is further illustrated in the next section where a few examples of remote relationships detected using AlignHUSH are presented.

### Examples of remote relationships discovered using AlignHUSH procedure

**Figure 3:**
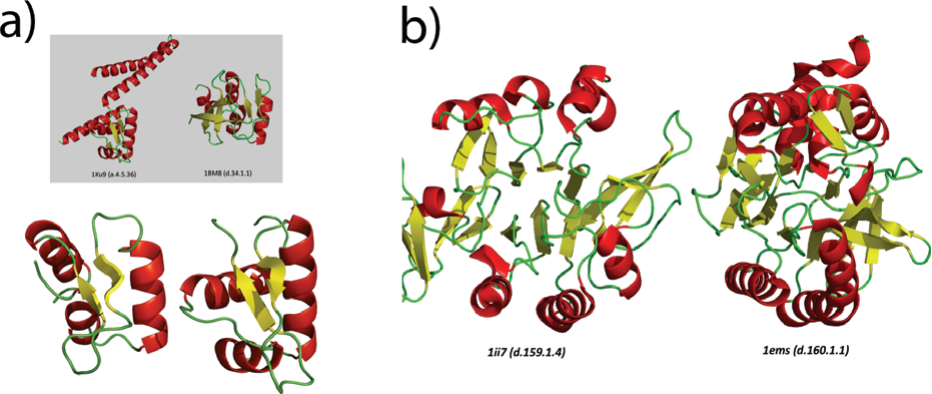
**Examples of two pairs of proteins with structural similarity between profiles that can be considered as false positives according to SCOP definition**. **A) **two proteins belonging to two different SCOP folds. The similarity in structure is evident from the figure and is also noted in the SCOP database. The structure on the right is 1KU9, N terminal part and the structure on left is 1BM8 (winged helix domain). The inset shows the full length proteins and the foreground picture shows the superposition of the part of each protein suggested to be homologous by AlignHUSH. **B) **two proteins belonging to different SCOP folds in the same class. Visual inspection does not seem to bring out the similarity between the two proteins and perhaps this is the reason why they are classified into two different folds by SCOP. The DALI Z score between the two proteins is around 8.0 covering 150 residues with an RMSD of 3.3 Å.

In the current analysis, relationships if identified between two SCOP families belonging to different SCOP folds (which include different SCOP classes) have been considered false positives. But the structural similarity between proteins involved in many such relationships is striking and has also been mentioned in SCOP. One such relationship is between the SCOP family DNA-binding domain of Mlu1-box binding protein MBP1 (SCOP code d.34.1.1) and the family DNA-binding protein Mj223 (SCOP code a.4.5.36, SCOP superfamily winged helix). The two families are from different classes but the method suggests similarity between the N-terminal part of Mlu1-box binding protein 1 and the Winged helix fold domain. Seven other families belonging to the winged helix superfamily are also found as hits with significant E-values strengthening the suggestion of homology between the N-terminal fragment of MBP1 protein and the winged helix superfamily. Interestingly, SCOP database mentions that the MBP1 family proteins have topological similarity to winged helix domains, but they are in different classes according to SCOP. Comparison of structures of regions of suggested similarity in two proteins (1KU9 and 1BM8) belonging to the two SCOP families, was done using DALI [37] and the structures are given in Figure 3a (the alignment is provided in Additional File 3). The structures in the figure suggest that there is a significant structural similarity between the proteins. AlignHUSH, as it is basically sequence-based, suggests that the structural similarity might be due to common evolutionary ancestry.

Another example (given in Figure 3b) of structural similarity is between the families Nitrilase (SCOP code d.160.1.1, Superfamily carbon-nitrogen hydrolase) and DNA double strand break repair nuclease family (SCOP code d.159.1.4, Superfamily metallo-dependent phosphatases). In this case too, SCOP mentions that the two families (in different folds of the same class), might have topological similarities. The Nitrilase family protein from *C.elegans *(PDB code 1ems) finds a homolog of known structure with Z score of 8.0 in the DALI database. The structural classification schemes do not provide indication of divergent evolution reliably at the SCOP fold level or above. Thus, if a method such as AlignHUSH finds a hit to a profile that is present in a different SCOP fold or SCOP class, it cannot always be considered a genuine false positive. The structural similarity and evolutionary signatures might still be present in such proteins, and since the alignment accuracy of the profile-profile methods is high, such cases must be treated manually (the alignment for the proteins in Figure 3b is given in Additional file 4 to illustrate this point).

**Figure 4:**
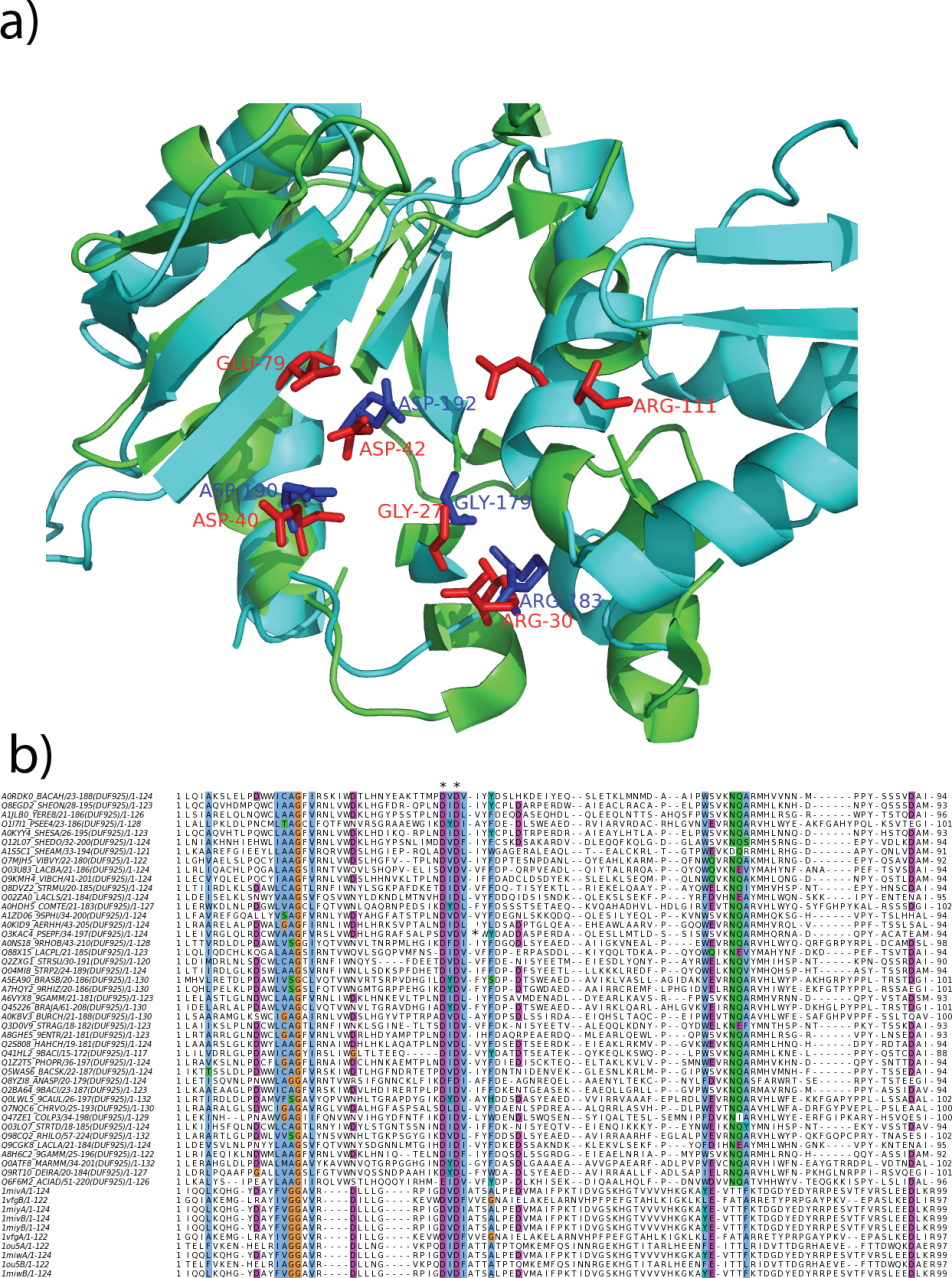
**Assessment of function annotation transfer between the DUF925 family and the Nucleotidyl transferase family (d.218.1.4)**. a) The structural alignment between two proteins in the SCOP superfamily of Nucleotidyltransferase, 1miv (shown in blue) and 7icq (shown in green). The active site residues in 1miv are shown in red, and the active site residues of 7icq are shown in blue, both in the stick format. The nucleotide binding residues in 1miv (Asp40 and Asp42) are seen to be aligned with the nucleotide binding residues of 7icq (Asp190 and Asp192). The active site is mostly conserved, but there are some differences between the two proteins which could perhaps explain the different substrates and mechanism employed by the two proteins. b) The alignment between the DUF925 family proteins and the SCOP family d.218.1.4 proteins. The alignment shows that the Aspartate residues important for binding nucleotides are conserved across the two families, but the conservation of other active site residues is not observed. Alignment figure generated using Jalview [39] and structure figure generated using PyMOL [40].

The AlignHUSH procedure can be useful for annotation of families of proteins with as yet unknown function (the DUF families of Pfam database). The DUF925 family is an example of a family of proteins with unknown function for which AlignHUSH has enabled a putative functional annotation. This family has been recognized as related to the d.218.1 (Nucleotidyltransferase superfamily in SCOP database) using AlignHUSH with d.218.1.4 family (Poly A polymerase head domain-like). There are multiple families in this superfamily and the active site residues participating in the catalytic function may not be conserved across different families in this SCOP superfamily. For example, two proteins with PDB codes 1miv (tRNA CCA-adding enzyme, head domain) and 7icq (DNA polymerase beta-like), are both classified into the nucleotidyltransferase superfamily but their active sites are not exactly superposable (see Figure 4a).

The Aspartate residues that bind to Mg^2+ ^ion are conserved in the two proteins shown in Figure 4a, but the catalytic Arginine residue in 1miv is not conserved in the 7icq protein. Thus, the conservation of active site residues is not observed in this SCOP superfamily although it must be pointed out that each of the proteins in this superfamily binds to a Nucleotide and Mg^2+^, and hence the relationship of the DUF925 family to this SCOP superfamily suggests that the DUF 925 family proteins could participate in a nucleotidyl tranferase reaction, but the substrate and the mechanism of catalysis may be different than that in the existing SCOP families in this superfamily. The alignment of the DUF925 proteins with the top hit in the SCOP superfamily is given in Figure 4b, and shows conservation of some residues. The residues important for binding the Mg^2+ ^ion in the SCOP family are the two Aspartate residues marked in the alignment which align with Aspartate residues in the DUF family and a glutamate residue which has been substituted with an Aspartate or a Glutamate in most proteins in the DUF family and a serine residue in some proteins in the DUF family sequences. Thus, it seems likely that the DUF925 family adopts the same fold as the proteins in the Nucleotidyltransferase superfamily and the partial conservation of some residues important for binding the catalytically active metal ion suggests that the proteins in this DUF family could participate in a reaction similar to the Nucleotidyltransferase reaction. A more detailed mutagenesis experiments needs to be carried out to explore in detail the role of these proteins of this DUF family.

## Discussion

The method developed has been demonstrated to have better sensitivity than currently existing methods such as HHSearch and PRC leading to an increase of around 7% and 3% respectively when assessed using 1.69 release of SCOP database. The increase in sensitivity when the latest SCOP database is used is 4% and 6% with respect to HHSearch and PRC respectively. The alignment accuracy is also comparable or better than that of HHSearch and PRC (average alignment accuracy around 50% for all methods). However, there are certain issues which point out that the method developed can in principle have even better performance than it has in its current form. One of the major improvements can be in statistical evaluation. Currently there are no models analogous to that for local sequence alignments (Karlin-Altschul statistics), for profile-profile alignments. It is difficult to give a theoretical explanation for the gaps present in profile-profile alignments. Does it mean that all sequences in one profile have a tendency to have inserts at that position or does it mean that there are no homologous regions in the other profile? These issues can be resolved if a detailed model of evolution which goes beyond family level of sequence similarity is generated. A lot of work in recent years seems to be moving in this direction but a clear answer is yet to emerge. Thus, at present ad hoc methods like curve-fitting need to be done to interpret the alignment scores statistically. Some of the previous works in the area have used E-values derived from PSI-BLAST like calculations [18] and others have devised novel techniques to estimate statistical significance [22].

Although it has been demonstrated that the alignment accuracy of AlignHUSH is quite high, it must be pointed out that the results must be carefully scrutinized. The assessment using BaliBASE reference alignments points out that the alignments bring the topologically equivalent secondary structures in register most of the time, and also align specific residues correctly 50% of the cases studied(in the modest number of alignments in the BaliBASE datasets). If the BaliBASE alignments are a true representative of the distant relationships existing in the structural universe, then the alignment accuracy estimated in the current work can be extended to all the alignments produced by AlignHUSH. Currently, there is no data to prove that there is a bias in the BaIiBASE alignment datasets, and hence the alignment accuracy values can be taken as truly representative of all the alignments produced by AlignHUSH. Other measures of alignment accuracy such as the GDT_TS score have been proposed and used in earlier work [22,25]. The GDT_TS score was developed to evaluate the match between a modeled structure and a real structure, and thus cannot handle large inserts and deletions very accurately. Moreover, the structural alignment between divergent proteins cannot be automatically generated with high reliability. Hence the applicability of the GDT_TS scores to remote structural similarities is not proved beyond doubt and hence, these scores have not been used in the current work.

The sensitivity of the developed method is quite high but it could in theory be higher for purely sequence based profiles. One of the major stumbling blocks in generation of profiles is the low number of sequences present in a family. For example, close to 90% of families in SCOP are composed of two or three proteins. In such a case, estimation of conservation becomes very difficult since almost every position might seem to be conserved. It is made more difficult if the sequence identity between the few proteins in the family is quite high. On the other hand, if sequence identity is very low on average in a family, then getting a high quality MSA becomes very difficult [38]. So, at present the quality of profiles generated for such divergent families in either SCOP or Pfam databases might be low, and hence these families may not be performing very well in the current assessment. This is also reflected in the fluctuations in the sensitivity values when using two different databases for all three methods.

## Conclusions

A method for aligning profile HMMs of protein sequences has been developed. The algorithm takes into account three kinds of information from the sequence alignment in addition to conservation of amino acids at a position. The additional information used are the predicted secondary structure information, hydrophobicity of the aligned residues, and the information present in neighboring columns. Assessment of sensitivity and error-rate in a large database of proteins of know structure (SCOP), revealed that the sensitivity of our method is more than that of currently existing methods. The alignments provided by AlignHUSH have comparable accuracy or slightly better accuracy than currently existing methods. The coverage of alignment on the profiles usually covers almost the entire length of the profile, and thus alignments generated by AlignHUSH can be used for function/structure annotation. A web-server incorporating the algorithm has been developed and is publicly available at http://crick.mbu.iisc.ernet.in/~alignhush/

## Competing interests

The authors declare that they have no competing interests.

## Authors' contributions

NS conceived of the study, and participated in its design and coordination and helped to draft the manuscript. OK implemented the method and performed the analysis of the results obtained using the method. Both the authors read and approved the final manuscript.

## Supplementary Material

Additional file 1 **Sensitivity at the fold level**. Sensitivity and error-rate values with true positives defined as SCOP families in the same fold. Click here for file

Additional File 2 **Sensitivity using latest SCOP release**. Sensitivity and error-rate values of HHSearch, PRC and AlignHUSH using the latest SCOP release. Click here for file

Additional file 3 **Alignment of two structurally similar families**. Sequence alignment of winged helix domain protein (pdb:1ku9) and DNA binding Mlu1 box protein generated using AlignHUSH. Click here for file

Additional file 4 **Alignment of two structurally similar families**. Sequence alignment of NIT-FHIT protein family and DNA double strand break repair family generated using AlignHUSH. Click here for file

## References

[B1] Pei J Multiple protein sequence alignment Curr Opin Struct Biol 2008 18 382 386 10.1016/j.sbi.2008.03.007 18485694

[B2] Moult J A decade of CASP: progress, bottlenecks and prognosis in protein structure prediction Curr Opin Struct Biol 2005 15 285 289 10.1016/j.sbi.2005.05.011 15939584

[B3] Bhadra R Srinivasan N Pandit SB A new domain family in the superfamily of alkaline phosphatases In Silico Biol 2005 5 379 387 16268782

[B4] Kuzniar A van Ham RC Pongor S Leunissen JA The quest for orthologs: finding the corresponding gene across genomes Trends Genet 2008 24 539 551 10.1016/j.tig.2008.08.009 18819722

[B5] Altschul SF Madden TL Schaffer AA Zhang J Zhang Z Miller W Lipman DJ Gapped BLAST and PSI-BLAST: a new generation of protein database search programs Nucleic Acids Res 1997 25 3389 3402 10.1093/nar/25.17.3389 9254694 PMC146917

[B6] Stojmiroviæ A Gertz EM Altschul SF Yu YK The effectiveness of position- and composition-specific gap costs for protein similarity searches Bioinformatics 2008 24 i15 i23 10.1093/bioinformatics/btn171 18586708 PMC2718649

[B7] Jaroszewski L Rychlewski L Li Z Li W Godzik A FFAS03: a server for profile--profile sequence alignments Nucleic Acids Res 2005 33 W284 W288 10.1093/nar/gki418 15980471 PMC1160179

[B8] Sadreyev R Grishin N COMPASS: a tool for comparison of multiple protein alignments with assessment of statistical significance J Mol Biol 2003 326 317 336 10.1016/S0022-2836(02)01371-2 12547212

[B9] Eddy SR Profile hidden Markov models Bioinformatics 1998 14 755 763 10.1093/bioinformatics/14.9.755 9918945

[B10] Krogh A Brown M Mian IS Sjolander K and Haussler D Hidden Markov models in computational biology: Applications to protein modeling J Mol Biol 1994 235 1501 1531 10.1006/jmbi.1994.1104 8107089

[B11] Madera M Gough J A comparison of profile hidden Markov model procedures for remote homology detection Nucleic Acids Res 2002 30 4321 4328 10.1093/nar/gkf544 12364612 PMC140544

[B12] Sjölander K Karplus K Brown M Hughey R Krogh A Mian IS Haussler D Dirichlet mixtures: a method for improved detection of weak but significant protein sequence homology Comput Appl Biosci 1996 12 327 345 8902360 10.1093/bioinformatics/12.4.327

[B13] Gribskov M Profile analysis Methods Mol Biol 1994 25 247 266 8004170 10.1385/0-89603-276-0:247

[B14] Mehta PK Argos P Barbour AD Christen P Recognizing very distant sequence relationships among proteins by family profile analysis Proteins 1999 35 387 400 10.1002/(SICI)1097-0134(19990601)35:4<387::AID-PROT2>3.0.CO;2-V 10382666

[B15] Marti-Renom MA Madhusudhan MS Sali A Alignment of protein sequences by their profiles Protein Sci 2004 13 1071 1087 10.1110/ps.03379804 15044736 PMC2280052

[B16] Edgar RC Sjölander K COACH: profile-profile alignment of protein families using hidden Markov models Bioinformatics 2004 20 1309 1318 10.1093/bioinformatics/bth091 14962937

[B17] Yona G Levitt M Within the twilight zone: a sensitive profile-profile comparison tool based on information theory J Mol Biol 2002 315 1257 1275 10.1006/jmbi.2001.5293 11827492

[B18] Soding J Protein homology detection by HMM-HMM comparison Bioinformatics 2005 21 951 960 10.1093/bioinformatics/bti125 15531603

[B19] Anand B Gowri VS Srinivasan N Use of multiple profiles corresponding to a sequence alignment enables effective detection of remote homologues Bioinformatics 2005 21 2821 2826 10.1093/bioinformatics/bti432 15817691

[B20] Sandhya S Chakrabarti S Abhinandan KR Sowdhamini R Srinivasan N Assessment of a rigorous transitive profile based search method to detact remotely similar proteins J Biomol Str Dyn 2005 23 283 298 10.1080/07391102.2005.10507066 16218755

[B21] Friedberg I Kaplan T Margalit H Evaluation of PSI-BLAST alignment accuracy in comparison to structural alignments Protein Sci 2000 9 2278 2284 10.1110/ps.9.11.2278 11152139 PMC2144484

[B22] Sadreyev RI Grishin NV Accurate statistical model of comparison between multiple sequence alignments Nucleic Acids Res 2008 36 2240 2248 10.1093/nar/gkn065 18285364 PMC2367703

[B23] Johnson LS Eddy SR Portugaly E Hidden Markov model speed heuristic and iterative HMM search procedure BMC Bioinformatics 2010 11 431 10.1186/1471-2105-11-431 20718988 PMC2931519

[B24] Madera M Profile Comparer: a program for scoring and aligning profile hidden Markov models Bioinformatics 2008 24 2630 2631 10.1093/bioinformatics/btn504 18845584 PMC2579712

[B25] Wang Y Sadreyev RI Grishin NV PROCAIN: protein profile comparison with assisting information Nucleic Acids Res 2009 37 3522 3530 10.1093/nar/gkp212 19357092 PMC2699500

[B26] Tang CL Xie L Koh IY Posy S Alexov E Honig B On the role of structural information in remote homology detection and sequence alignment: new methods using hybrid sequence profiles J Mol Biol 2003 334 1043 1062 10.1016/j.jmb.2003.10.025 14643665

[B27] Biegert A Söding J Sequence context-specific profiles for homology searching Proc Natl Acad Sci USA 2009 106 3770 3775 10.1073/pnas.0810767106 19234132 PMC2645910

[B28] Gough J Chothia C SUPERFAMILY: HMMs representing all proteins of known structure. SCOP sequence searches, alignments and genome assignments Nucleic Acids Res 2002 30 268 272 10.1093/nar/30.1.268 11752312 PMC99153

[B29] Wilson D Pethica R Zhou Y Talbot C Vogel C Madera M Chothia C Gough J SUPERFAMILY-- sophisticated comparative genomics, data mining, visualization and phylogeny Nucleic Acids Res 2009 37 D380 D386 10.1093/nar/gkn762 19036790 PMC2686452

[B30] Andreeva A Howorth D Chandonia J.-M Brenner SE Hubbard TJ Chothia C Murzin AG Data growth and its impact on the SCOP database: new developments Nucleic Acids Res 2008 36 D419 D425 18000004 10.1093/nar/gkm993 PMC2238974

[B31] Roseman MA Hydrophilicity of polar amino acid side-chains is markedly reduced by flanking peptide bonds J Mol Biol 1988 200 513 522 10.1016/0022-2836(88)90540-2 3398047

[B32] Jones DT Protein secondary structure prediction based on position-specific scoring matrices J Mol Biol 1999 292 195 202 10.1006/jmbi.1999.3091 10493868

[B33] Karlin S Altschul SF Methods for assessing the statistical significance of molecular sequence features by using general scoring schemes Proc Natl Acad Sci USA 1990 87 2264 2268 10.1073/pnas.87.6.2264 2315319 PMC53667

[B34] Karlin S Altschul SF Applications and statistics for multiple high-scoring segments in molecular sequences Proc Natl Acad Sci USA 1993 90 5873 5877 10.1073/pnas.90.12.5873 8390686 PMC46825

[B35] Eddy SR A probabilistic model of local sequence alignment that simplifies statistical significance estimation PLoS Comput Biol 2008 4 e1000069 10.1371/journal.pcbi.1000069 18516236 PMC2396288

[B36] Thompson JD Koehl P Ripp R Poch O BAliBASE 3.0: latest developments of the multiple sequence alignment benchmark Proteins 2005 61 127 136 10.1002/prot.20527 16044462

[B37] Holm L Sander C Protein structure comparison by alignment of distance matrices J Mol Biol 1993 233 123 138 10.1006/jmbi.1993.1489 8377180

[B38] Do CB Katoh K Protein multiple sequence alignment Methods Mol Biol 2008 484 379 413 10.1007/978-1-59745-398-1_25 18592193

[B39] Waterhouse AM Procter JB Martin DMA Clamp M Barton GJ Jalview Version 2 - a multiple sequence alignment editor and analysis workbench Bioinformatics 2009 25 1189 119 10.1093/bioinformatics/btp033 19151095 PMC2672624

[B40] DeLano WL The PyMOL Molecular Graphics System DeLano Scientific LLC, Palo Alto, CA, USA http://www.pymol.org

