# Supplementary material for: AlignHUSH: Alignment of HMMs using structure and hydrophobicity information
Source: BMC Bioinformatics. 2011 Jul 5;12:275. doi: 10.1186/1471-2105-12-275 (PMC3228556; doi:10.1186/1471-2105-12-275)
Supplement: Additional file 1 — Sensitivity at the fold level. Sensitivity and error-rate values with true positives defined as SCOP families in the same fold. [file 1471-2105-12-275-S1.DOC]

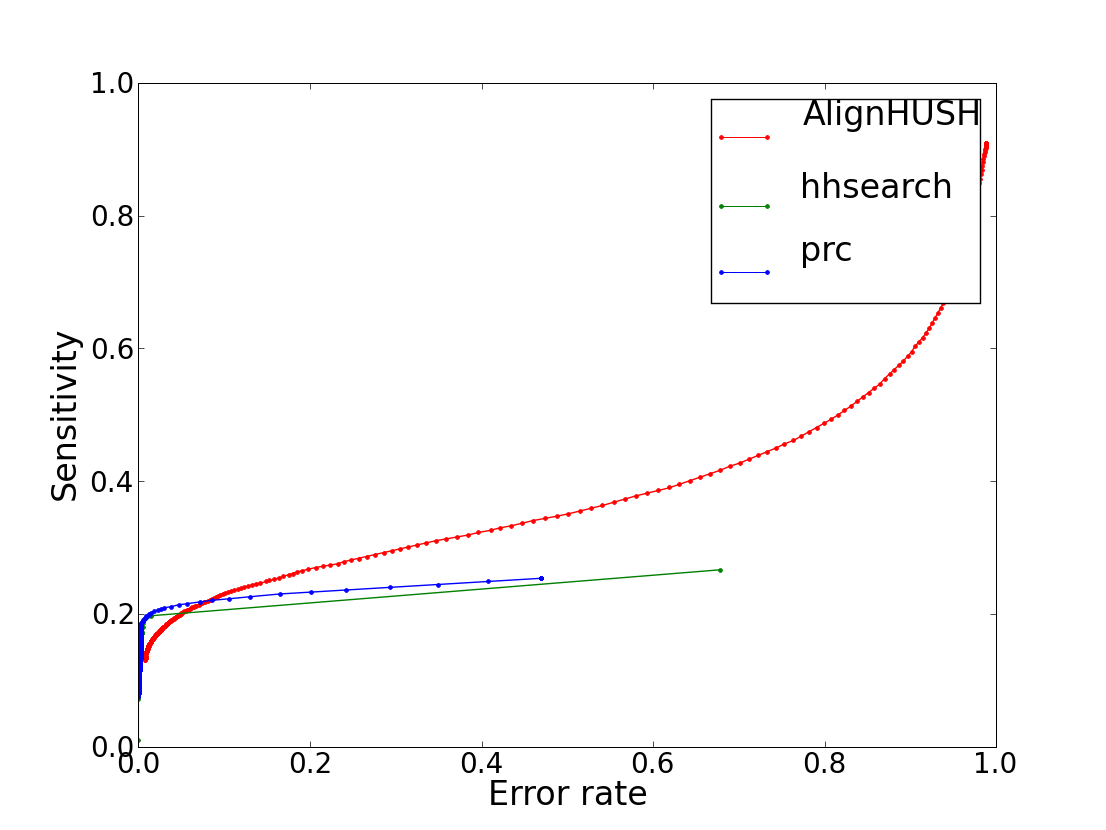


Additional file 1: Comparison of performance of AlignHUSH method to that of HHSearch and PRC. The sensitivity rate in this figure is calculated as the ratio of SCOP families in the same SCOP fold found to be related by each of the methods. The error rate has been calculated as in Fig 1a.
