# Supplementary material for: AlignHUSH: Alignment of HMMs using structure and hydrophobicity information
Source: BMC Bioinformatics. 2011 Jul 5;12:275. doi: 10.1186/1471-2105-12-275 (PMC3228556; doi:10.1186/1471-2105-12-275)
Supplement: Additional File 2 — Sensitivity using latest SCOP release. Sensitivity and error-rate values of HHSearch, PRC and AlignHUSH using the latest SCOP release. [file 1471-2105-12-275-S2.DOC]

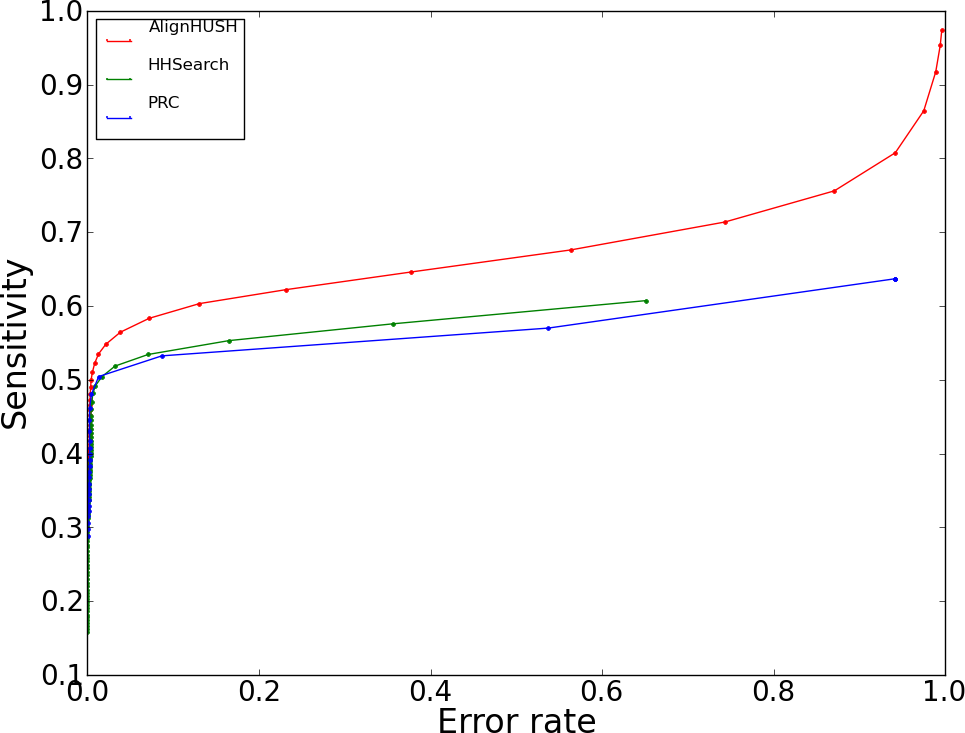


Additional file 2: Comparison of performance of AlignHUSH method to that of HHSearch and PRC on the latest SCOP release (SCOP ver 1.75). The sensitivity rate and error rate have been calculated as in Fig 1a. The sensitivity of AlignHUSH at 10% error rate is ~59, of HHSearch is ~54 and that of PRC is ~53. Note that the sensitivity values for AlignHUSH and PRC were based on Superfamily.org profiles in HMMER3.0 format whereas the HHSearch values are from the HMMs downloaded from HHSearch web-site corresponding to the Superfamily.org database version.
