# Supplementary material for: AlignHUSH: Alignment of HMMs using structure and hydrophobicity information
Source: BMC Bioinformatics. 2011 Jul 5;12:275. doi: 10.1186/1471-2105-12-275 (PMC3228556; doi:10.1186/1471-2105-12-275)
Supplement: Additional file 3 — Alignment of two structurally similar families. Sequence alignment of winged helix domain protein (pdb:1ku9) and DNA binding Mlu1 box protein generated using AlignHUSH. [file 1471-2105-12-275-S3.DOC]

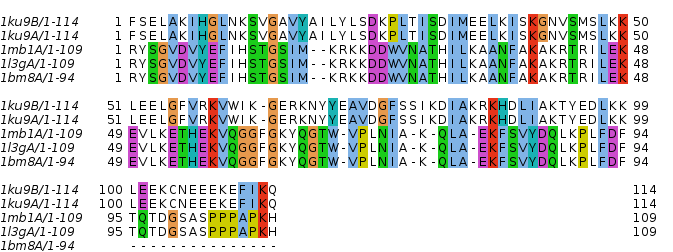


Additional file 3: Alignment between the winged helix protein 1ku9 and three proteins in the d.34.1.1 family of SCOP (DNA binding Mlu1 box protein). The residues are colored according to the code used in ClustalX. It can be seen that between the two families, some amino acids are conserved. The figure was generated using Jalview.
