# Supplementary material for: AlignHUSH: Alignment of HMMs using structure and hydrophobicity information
Source: BMC Bioinformatics. 2011 Jul 5;12:275. doi: 10.1186/1471-2105-12-275 (PMC3228556; doi:10.1186/1471-2105-12-275)
Supplement: Additional file 4 — Alignment of two structurally similar families. Sequence alignment of NIT-FHIT protein family and DNA double strand break repair family generated using AlignHUSH. [file 1471-2105-12-275-S4.DOC]

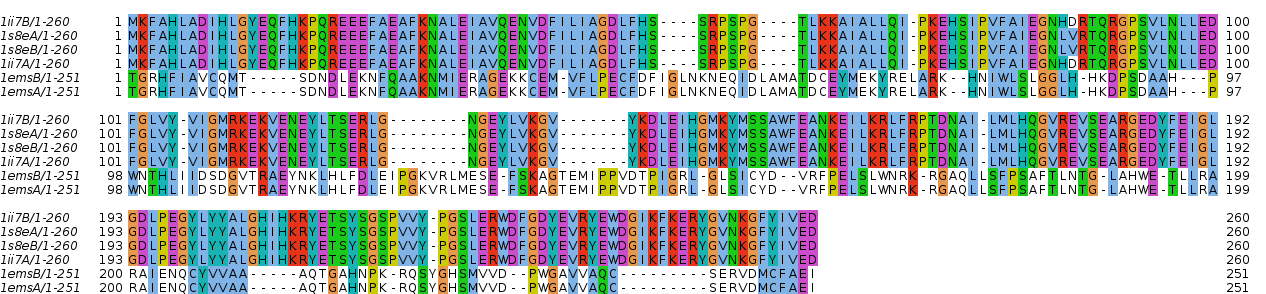


**Additional file 4:** The figure shows the alignment between the NIT-FHIT protein and close homologues and 1ems of DNA double strand break repair family. The residues are colored according to the ClustalX and the figure shows conservation of amino acids in many alignment positions. High similarity at the level of 3-D structures and sequence-profiles of the two protein families strongly points to an evolutionary link between the two families. The figure was generated using Jalview.
